# Inclined angles of acetabular quadrilateral plate: digital measurement and clinical application of the new anatomical concept

**DOI:** 10.1186/s13018-023-04143-3

**Published:** 2023-09-25

**Authors:** Xiaofeng Chen, Haiyang Wu, Kunming Cheng, Ximing Liu, Xianhua Cai

**Affiliations:** 1 Department of Orthopaedic Surgery, Yangxin People’s Hospital, Yangxin, 435200 Hubei China; 2 Department of Clinical Medicine, Graduate School of Tianjin Medical University, Tianjin, 301700 China; 3 Department of Intensive Care Unit, The Second Affiliated Hospital of Zhengzhou University, Zhengzhou, Henan China; 4 Department of Orthopaedic Surgery, General Hospital of Central Theater Command, Wuhan, 430064 Hubei China; 5 Department of Orthopaedic Surgery, South China Hospital Affiliated to Shenzhen University, Shenzhen, 518111 Guangdong China

**Keywords:** Inclined angle, Acetabular fracture, Quadrilateral plate, Digital measurements

## Abstract

**Purpose:**

Matta scoring standard is one of the most frequently used postoperative imaging evaluations for acetabular fracture reduction, but has obvious shortcomings. This study, for the first time, proposed the concept of inclined angles of acetabular quadrilateral plate. The purpose of this study was to investigate the normal range of the inclined angles in adults by digital measurement and explore the feasibility of using them to evaluate the reduction quality of acetabular quadrilateral fractures after surgery.

**Methods:**

Firstly, the pelvic CT three-dimensional reconstruction data of 40 healthy adults including 20 males and 20 females were collected. The normal range of the anterior, middle, and posterior inclined angles were measured via Mimics software. Secondly, a modified Matta criteria that combined the classic Matta criteria and evaluation criteria of the inclined angles was proposed. And we classified modified Matta criteria into three grades including excellent, good and poor. Finally, a total of 125 cases with quadrilateral plate fractures was included and the postoperative CT data were analyzed by using both the classic Matta criteria and our modified Matta criteria. Then, the accuracy and consistency of both criteria to evaluate postoperative hip function was investigated.

**Results:**

The average anterior inclined angle: male (97.11° ± 2.59°), female (90.63° ± 2.09°); middle inclined angle: male (105.57° ± 1.93°), female (100.64° ± 2.46°); and posterior inclined angle: male (112.62° ± 2.54), female (106.37° ± 2.53°). Whether in males or in females, the anterior, middle, and posterior inclined angles showed a progressively increasing trend. All the three inclined angles in males were all significantly larger than those in females (*p* < 0.05). Among 125 cases with quadrilateral plate fractures, 101 cases (80.8%) were graded as excellent, 18 cases (14.4%) as good, and 6 cases (4.8%) as poor according to the classic Matta criteria. While based on modified Matta criteria, there were excellent in 37 cases (29.6%), good in 76 cases (60.8%), and poor in 12 cases (9.6%). According to the Harris hip score system, the functional outcomes were excellent in 59 cases (47.2%), good in 26 cases (20.8%), fair in 24 cases (19.2%), and poor in 16 cases (12.8%). Our results showed that among the cases evaluated as excellent according to the classic Matta criteria and modified Matta criteria, the excellent-to-good rates of hip function were 70.3% and 78.4%, respectively. And among the cases evaluated as poor according to the modified Matta criteria, the fair-to-poor rate of hip function was 75%, while this value was 50% for classic Matta criteria. Both differences were statistically significant (*p* < 0.05).

**Conclusion:**

Inclined angles of the quadrilateral plate could be used to assess the quality of fracture reduction and provide a basis for evaluating the rotational displacement of fracture blocks in the quadrilateral plate, which compensates the shortage of classic Matta criteria.

## Introduction

The structural integrity of the acetabulum serves as the foundation for maintaining hip joint stability and normal function. As for acetabular fractures, anatomical reduction is essential for obtaining biomechanical stability and functional recovery. Acetabular fractures could cause step or fissure displacement, which increases the pressure between the femoral head and acetabular articular surface, and leads to altered stress distribution in the hip joint, ultimately resulting in the development of post-traumatic arthritis [1, 2]. At present, the Matta scoring standard is one of the most frequently used postoperative imaging evaluations for acetabular fracture reduction [3]. According to the residual displacement distance of the fracture, the quality of reduction was judged as excellent, good or poor. The advantage of this scoring criteria is that the evaluation process is quick and convenient, and could be performed using postoperative imaging such as X-rays or three-dimensional (3D) CT reconstructions. However, although the validity of the Matta criterion has been extensively verified, it has obvious shortcomings because it only deals with fracture alignment and does not take into account fracture rotational displacement [4, 5].

The acetabular quadrilateral plate forms the medial wall of the acetabulum and plays a critical role in preventing the femoral head from dislocating into the pelvis [6]. According to Letournel-Judet classification system, in addition to simple anterior and posterior wall fractures, all the other eight fracture classifications such as anterior column posterior hemi-transverse (ACPHT) fractures, T-shape fractures, both-column fractures, and so on, will inevitably involve quadrilateral plate [7, 8]. Thus, quadrilateral plate is an important anatomic structure for fracture reduction and some researchers even proposed separate classifications of quadrilateral plate fractures. The quadrilateral plate is tilted relative to the pelvic cross-section structure, but it has not been reported in the literature before. Furthermore, rotational displacement of the quadrilateral plate is able to alter the physiological weight-bearing line of the lower limb and accelerate degenerative changes in the affected hip and even knee joints [9]. Previous studies by our research group have found that Walid’s classification system, one fracture classification method that divided the quadrilateral plate fractures into four categories based on degree of comminution, was associated with the reduction quality and functional recovery [10]. Therefore, it is crucial to identify indicators that could evaluate the rotational displacement of the quadrilateral plate to further improve postoperative imaging evaluation. The definition of the inclined angle of quadrilateral plate has not been reported yet. Thus, this study defines it as the angle between the line connecting the bilateral ischial tuberosity or its parallel line and the bone surface of the quadrilateral plate.

In view of this, this study proposed to incorporate the assessment of the quadrilateral plate inclination angle based on the Matta criteria, which considered not only the fracture line alignment but also the angle alignment, thus making up for the shortcomings of the Matta criteria and proposing a modified version of the Matta standard. Furthermore, a group of pelvic CT 3D reconstructions of patients with acetabular quadrilateral fractures was collected and the reduction quality was evaluated by Matta criteria and modified Matta criteria (Matta criteria + quadrilateral plate inclination angle), respectively. We aimed to explore the feasibility of using this modified Matta criteria to evaluate the reduction quality of acetabular quadrilateral fractures after surgery.

## Materials and methods

### Digital measurement of inclined angle of acetabular quadrilateral plate

#### Collection of pelvic CT data

In this study, pelvic CT 3D reconstruction data were collected from our hospital. The inclusion criteria were complete pelvis without any lesion, anatomical abnormality, fracture of the healthy adults (aged between 30 and 60 years). Finally, a total of 40 pelvises was obtained. Among them, there were 20 male and 20 female cases with an age range of 31 to 58 years (mean, 44.2 years).

#### Pelvic CT scan and 3D reconstruction

The CT scanning parameters were set as follows: 120 kV, 300mAs, 0.75 mm layer thickness. All CT data were saved in the DICOM format. Then the images were exported from PACS and imported into Mimics software (Version 10.01; Materialise Inc., Leuven, Belgium). The threshold segmentation, region growth and mask editing functions were utilized to remove soft tissue, bilateral femurs, and sacrum to establish a clearer and complete 3D pelvis model.

#### Measurements of inclined angle

**Fig. 1:**
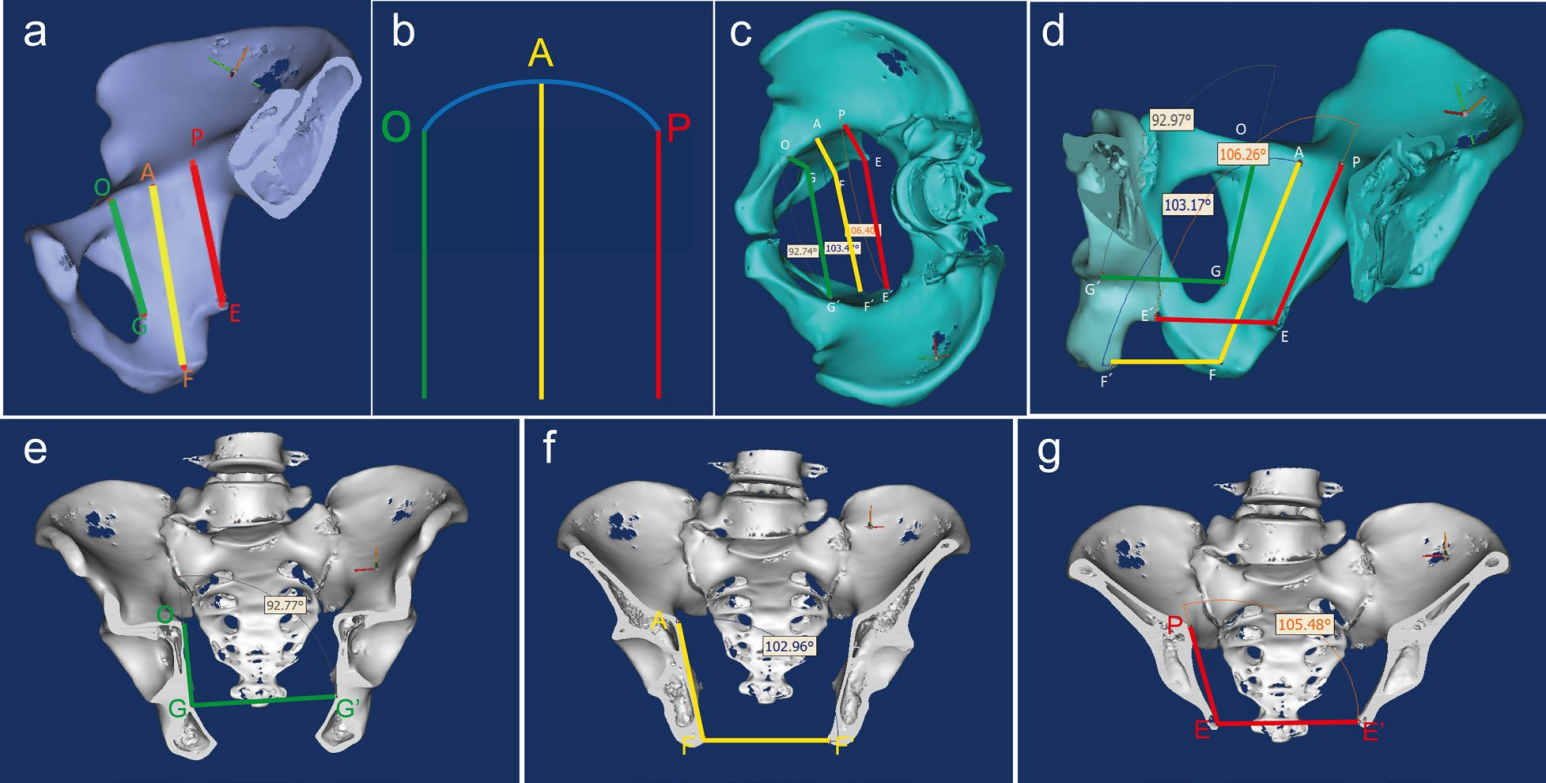
**a** The anterior (OG), middle (AF) and posterior (PE) inclined line of the quadrilateral plate; **b** schematic diagram of “three-line” divided quadrilateral plate; **c** Top view of the three inclined angles; **d** Oblique view of the three inclined angles. The lines “GG'”, “EE'”, and “FF'” were parallel to each other; **e** Coronal direction of anterior inclined angle∠OGG'; **f** Coronal direction of middle inclined angle∠AFF'; **g** Coronal direction of posterior inclined angle∠PEE'

As shown in Fig. 1a, b, three projected points of the quadrilateral plate on the pelvic boundary line were selected. A perpendicular line was drawn from the most anterior part of the acetabular rim to the tangent line of the pelvic boundary, and the intersection of the perpendicular line and the pelvic boundary line was defined as point “O”. Similarly, a perpendicular line was drawn from most posterior point of the acetabular rim to the tangent line of the pelvic boundary, and the intersection was defined as point “P”. Point “O” and point “P” represented the projections of the anterior and posterior edges of the acetabulum on the pelvic boundary line, respectively. Point “A” was the intersection of the perpendicular bisector of OP and the pelvic boundary line. The posterior inclined line of the quadrilateral plate was the “PE” line, which connected point “E” on the ischial spine to point “P”. The inclined line in the middle of the quadrilateral plate was the “AF” line, which connected point “F” on the ischial tuberosity to point “A”. A perpendicular line was drawn from point E to the tangent of the posterior border of the obturator foramen, and the intersection was “G”. The anterior inclined line of the quadrilateral plate was the “OG” line. The anterior and posterior inclined lines represented the anterior and posterior edges of the quadrilateral plate, respectively, and the middle inclined line passed through the bony surface of quadrilateral plate. Therefore, the anterior, middle, and posterior inclined lines could represent the overall trend of the quadrilateral plate.

As shown in Fig. 1c, “G'”, “E'”, and “F'” were the projections of points “G”, “E”, and “F” on the contralateral side of the pelvis, using the pelvic center sagittal plane as the axis of symmetry. “EE'” represented the line connecting both sides of the ischial spines, while “FF'” represented the line connecting both sides of ischial tuberosities. The lines “GG'”, “EE'”, and “FF'” were parallel to each other (Fig. 1d). The anterior, middle, and posterior inclined angles were composed of one side of the anterior, middle, and posterior inclined lines of “OG”, “AF”, and “PE”, and lines of “GG'”, “FF'”, and “EE'”. Therefore, the anterior, middle, and posterior inclined angles of the quadrilateral plate were ∠OGG', ∠AFF', and ∠PEE', respectively (Fig. 1e–g). Afterward, the angle measurement function in Mimics software could be used to directly measure the angle sizes of the three inclined angles in the all 40 pelvises.

### Clinical applications of inclined angle of acetabular quadrilateral plate

#### Clinical data

Patients with acetabular fracture underwent surgical treatments between June 2009 to June 2021 were retrospectively analyzed. Inclusion criteria: Patients with acetabular fractures involving the quadrilateral plate; (2) underwent surgical treatment; (3) aged between 20 and 75 years old; (4) closed fractures and underwent surgery within 2 weeks after injury. Exclusion criteria: (1) pathologic acetabular fractures; (2) patients with pre-existing abnormal conditions or avascular necrosis of the femoral head; (3) patients with severe cardiovascular or pulmonary dysfunction, or preoperative American Society of Anesthesiologists (ASA) grade ≥ IV; (4) patients with a history of mental disorders.

A total of 125 cases met the above criteria. Among them, there were 74 male and 51 female patients aged 22 ~ 71 years with an average age of 45.6 years. Fractures were located on the left side in 63 cases and on the right side in 62 cases. The causes of injury were traffic accidents in 82 cases, falls from height in 39 cases, and crushing injuries in 4 cases. Associated injuries included ipsilateral central dislocation of the femoral head in 9 cases, pelvic ring fractures in 11 cases, vertebral body fractures in 5 cases, fractures of the extremities in 13 cases, head trauma in 2 cases, abdominal organ injury in 7 cases, and sciatic nerve damage in 3 cases.

#### Surgical technique and perioperative management

Pelvic X-ray, CT scan and 3D reconstruction were routinely performed before surgery. All operations were performed with the same groups of senior surgeons using general anesthesia. A standard ilioinguinal approach was performed in all patients to gain the surgical fields of acetabular anterior column, the pelvic boundary, and the upper part of the quadrilateral plate. For patients combined with posterior wall fracture or difficult to reduce posterior column fracture, the Kocher–Langenbeck (K–L) approach was applied. As the fixation of fracture blocks in quadrilateral plate, a novel internal fixation system called the dynamic anterior plate-screw system for quadrilateral plates (DAPSQ) designed by our group was used. The detailed operation steps of DAPSQ were described in our previous reports [11, 12].

After surgery, prophylactic antibiotics were used regularly for 24 h. The drainage tube was removed when the drainage flow < 20 mL. Patients were encouraged to start non-weight-bearing rehabilitation such as passive and active ipsilateral hip flexion or extension motion after awakened from anesthesia. And then gradually progress to protected weight-bearing exercises and full-weight bearing. All patients underwent pelvic X-ray, CT scan and 3D reconstruction on the 3th ~ 5th postoperative day. After discharge, patients were requested to regular outpatient review at 1 month, 2 months, 3 months, 6 months, 1 year, and yearly thereafter. During the follow-up, fracture healing, radiographic progress, functional scores and complications were recorded.

### Evaluation criteria

#### Classic Matta criteria

Classic Matta grading scores were classified as excellent (< 1 mm displacement, anatomical reduction), good (1 ~ 3 mm displacement, satisfactory reduction), and poor (> 3 mm displacement, unsatisfactory reduction) based on millimeters of residual displacement evaluated from postoperative pelvic CT 3D reconstruction [3].

### Evaluation criteria of the inclined angles of quadrilateral plate

The quadrilateral plate could be divided into the first and second half areas. The abnormal alignment of the first half quadrilateral plate was reflected by the anterior and middle inclined angles, whereas the abnormal alignment of the second half quadrilateral plate was reflected by the middle and posterior inclined angles. Therefore, changes in the middle inclination angle were particularly important. If the anterior, middle, and posterior inclination angles were all within the normal range, it indicated that both the first and second half of the quadrilateral plate had good alignment. If the anterior, middle, and posterior inclination angles were are all out of the normal range, or only the middle inclination angle was abnormal, it indicated that both the first and second half of the quadrilateral plate had poor alignment. As illustrated in Table 1, we classified inclined angles of quadrilateral plate into three grades including excellent, good and poor. Excellent referred to all the inclined angles of anterior, middle, and posterior were within normal range. Poor referred to all the inclined angles of anterior, middle, and posterior were not within normal range, or only the middle inclined angle was not within normal range. While good included all remaining conditions. The normal ranges of inclined angles of anterior, middle, and posterior were acquired from the measurements of the normal 40 cases.

**Table 1:** Evaluation criteria of the anterior, middle, and posterior inclined angles of quadrilateral plate

Grade	Definition
Excellent	All the inclined angles of anterior, middle, and posterior were within normal range
Good	The anterior and middle inclined angles were within normal range, but the posterior inclined angle was not; The middle and posterior inclined angles were within normal range, but the anterior inclined angle was not; The middle inclined angle was within normal range, but the anterior and posterior inclined angle were not
Poor	All the inclined angles of anterior, middle, and posterior were not within normal range, or only the middle inclined angle was not within normal range

Normal range of anterior inclined angle: Male (95.94° ~ 98.32°); Female (89.69° ~ 91.61°)

Normal range of middle inclined angle: Male (104.68° ~ 106.38°); Female (99.55° ~ 101.78°)

Normal range of posterior inclined angle: Male (111.43° ~ 113.70°); Female (105.26° ~ 107.41°)

### Modified Matta criteria

Modified Matta criteria was the combination of classic Matta criteria and evaluation criteria of the inclined angles of quadrilateral plate. We also classified modified Matta criteria into three grades including excellent, good, and poor. The main definition is summarized in Table 2. For example, excellent indicated that classic Matta criteria was classified as excellent and evaluation criteria of inclined angles was also classified as excellent, whereas poor referred to classic Matta criteria classified as good or poor and evaluation criteria of inclined angles classified as poor, or classic Matta criteria classified as poor and evaluation criteria of inclined angles classified as good. While good included all remaining conditions.

**Table 2:** Evaluation criteria of the modified Matta criteria

Grade	Definition
Excellent	Classic Matta criteria was classified as excellent and evaluation criteria of inclined angles was also classified as excellent
Good	Classic Matta criteria was classified as excellent and evaluation criteria of inclined angles was also classified as good; Classic Matta criteria was classified as good and evaluation criteria of inclined angles was also classified as good; Classic Matta criteria was classified as excellent and evaluation criteria of inclined angles was also classified as poor; Classic Matta criteria was classified as poor and evaluation criteria of inclined angles was also classified as excellent Classic Matta criteria was classified as good and evaluation criteria of inclined angles was also classified as excellent
Poor	Classic Matta criteria was classified as poor and evaluation criteria of inclined angles was also classified as poor Classic Matta criteria was classified as good and evaluation criteria of inclined angles was also classified as poor Classic Matta criteria was classified as poor and evaluation criteria of inclined angles was also classified as good

### Harris hip score

The postoperative hip joint function was evaluated using the Harris hip score system, which takes into account factors such as joint pain, deformity, range of motion, ambulation, and activities of daily living. The evaluation criteria were as follows: excellent: 90 ~ 100 points; good: 80 ~ 89 points; fair: 70 ~ 79 points; poor: < 70 points. The excellent and good rates were calculated as the percentage of the sum number of cases rated as excellent and good to the total number of cases [13].

### Assessment method

The postoperative CT 3D reconstructions of 125 cases were evaluated by using both the classic Matta criteria and our modified Matta criteria. If the distribution of excellent, good, and poor postoperative hip function as evaluated by the modified Matta criteria is more accurate and consistent than the classic Matta criteria, it can be demonstrated that the modified Matta criteria is superior to the classic Matta criteria. For example, in the cases of Harris Hip Score rated as excellent, if the modified Matta criteria could grade more excellent cases than classic Matta criteria, or in the cases of Harris Hip Score rated as poor, if the modified Matta criteria could grade more poor cases, that means modified Matta criteria is more reliable to accurately predict the functional status after surgery.

### Statistical analysis

Statistical analysis was conducted by using the SPSS software (Version 19.0, Chicago, IL, USA). The continuous variables were firstly tested by Kolmogorov–Smirnov test for normal distribution and data with normal distribution were expressed as mean ± SD and non-normal distribution data were expressed as median (and interquartile range). Comparisons between two independent groups were conducted by independent sample *t* test. Categorical variables were presented as absolute (*n*) and relative (%) frequencies. Categorical data were tested using the chi-square test. A *p*-value of less than 0.05 was considered statistically significant.

## Results

### Comparison of male and female normal inclined angle of quadrilateral plate

**Fig. 2:**
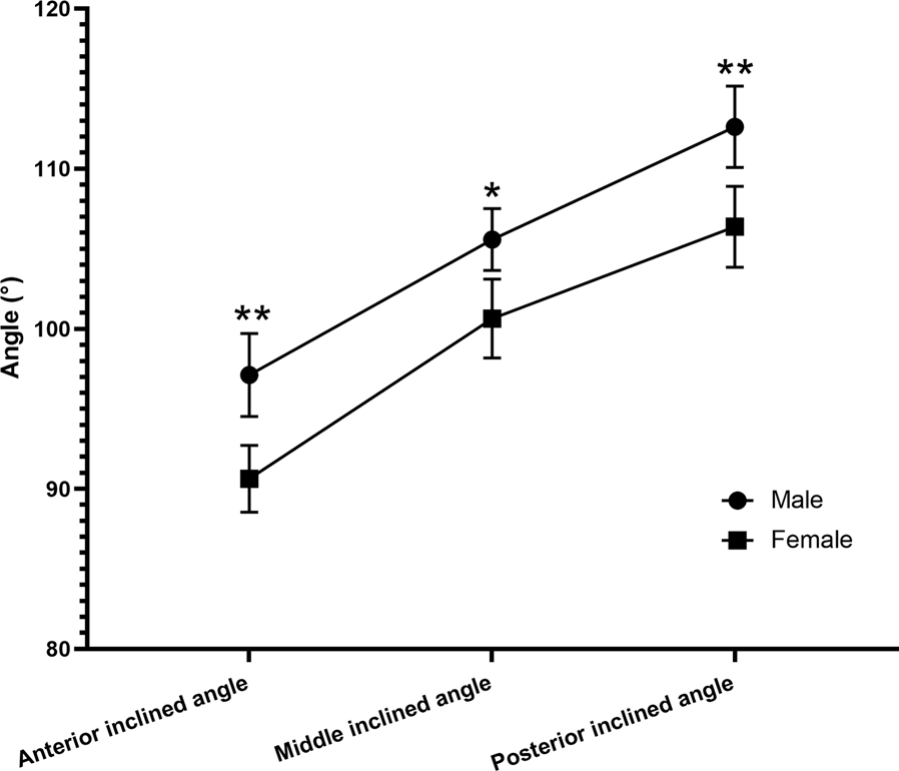
Comparison of male and female inclined angle of quadrilateral plate. *p < 0.05; **p < 0.01

The measurement results of the anterior, middle, and posterior inclined angle of quadrilateral plate for male and female are shown in Fig. 2. The normal anterior inclined angle of male population ranged from 95.94° to 98.32°, with an average angle of 97.11° ± 2.59°. While for female population, the normal anterior inclined angle ranged from 89.69° to 91.61°, with an average angle of 90.63° ± 2.09°. The normal middle inclined angle of male population ranged from 104.68° to106.38° (105.57° ± 1.93°), and female population ranged from 99.55° to 101.78° (100.64° ± 2.46°). The normal posterior inclined angle of male population ranged from 111.43° to 113.70° (112.62° ± 2.54°), and female population ranged from 105.26° to 107.41° (106.37° ± 2.53°). As can be seen, the anterior, middle, and posterior inclined angles in males were all significantly larger than those in females (*p* < 0.05). Meanwhile, whether in males or in females, the anterior, middle, and posterior inclined angles showed a progressively increasing trend. The average difference between the anterior and middle inclined angles in male was about 8°, and the average difference between the middle and posterior angles was about 7°. As for female, the average difference between the anterior and middle inclined angles was about 10°, while the average difference between the middle and posterior angles was about 6°.

### Reduction quality and functional scores of patients with acetabular fractures

According to the Harris hip score system, the functional outcomes were excellent in 59 cases (47.2%), good in 26 cases (20.8%), fair in 24 cases (19.2%), and poor in 16 cases (12.8%). Postoperative classic Matta grading scores showed that 101 cases (80.8%) were graded as excellent, 18 cases (14.4%) as good, and 6 cases (4.8%) as poor. According to our modified Matta criteria, there were excellent in 37 cases (29.6%), good in 76 cases (60.8%), and poor in 12 cases (9.6%). As shown in Table 3, among the 101 excellent cases evaluated by classic Matta criteria, there are 37, 34, and 30 cases rating as excellent, good, and poor, respectively, according to the evaluation criteria of the inclined angles. As for 18 good cases, 6, 5, and 7 cases rating as excellent, good, and poor, respectively. And in terms of the 6 poor cases, 1, 2, and 3 cases rating as excellent, good and poor, respectively.

**Table 3:** The distribution of excellent, good, and poor cases based on inclined angles evaluation criteria

Class Matta criteria	Excellent (*n* = 101)			Good (*n* = 18)			Poor (*n* = 6)		
Inclined Angles criteria	Excellent	Good	Poor	Excellent	Good	Poor	Excellent	Good	Poor
Number of cases	37 (36.6%)	34 (33.7%)	30(29.7%)	6(33.3%)	5(27.8%)	7(38.9%)	1(16.7%)	2(33.3%)	3(50%)

### Comparison of the classic Matta criteria and modified Matta criteria

**Fig. 3:**
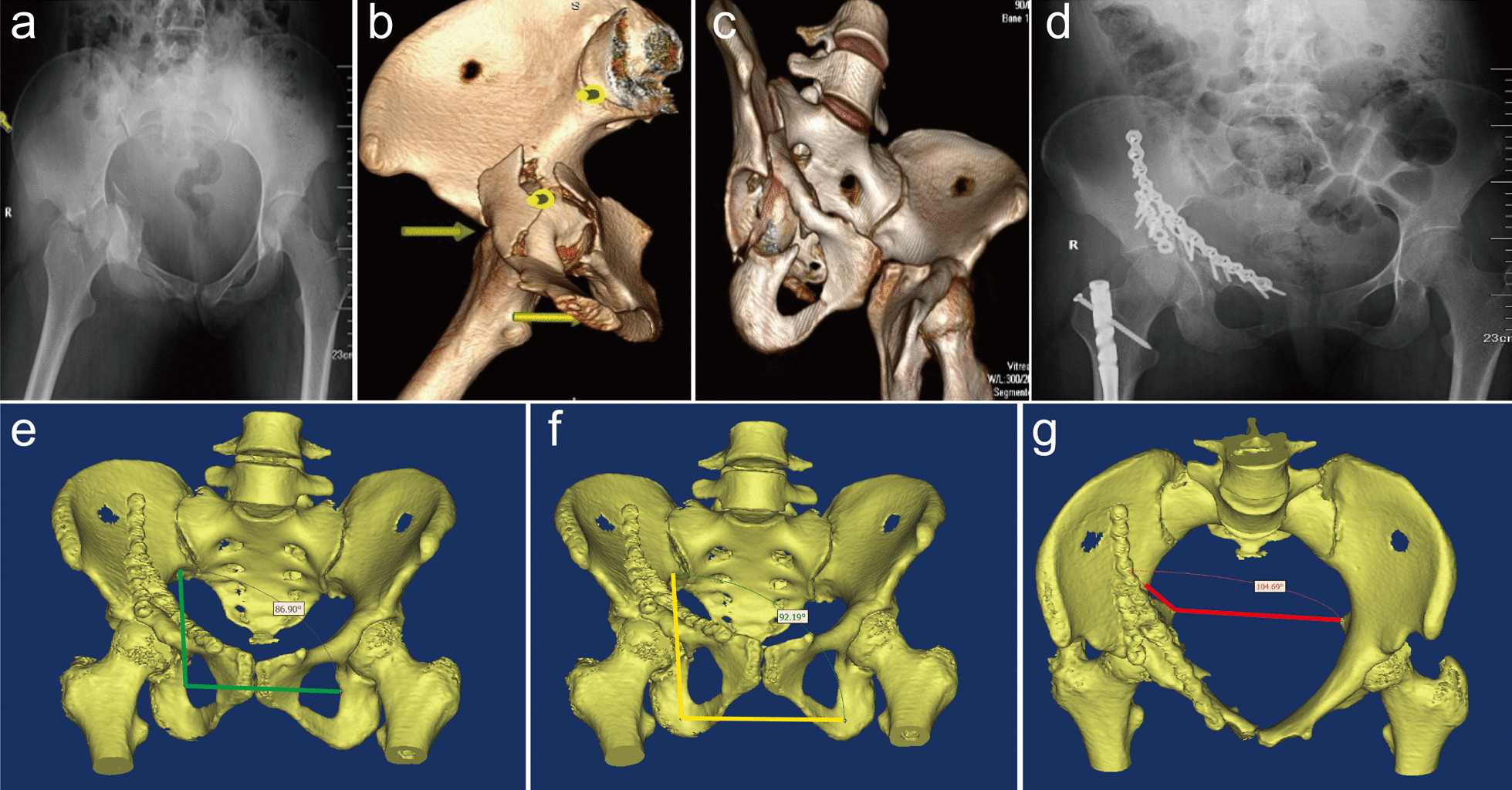
Typical case: A 23-year-old woman presented with T-shaped fracture of the right acetabulum (Letournel-Judet classification). Preoperative X-ray (**a**) and CT 3d reconstruction (**b**–**c**) showed significant fracture displacement of quadrilateral plate. Dynamic anterior plate-screw system for quadrilateral plates (DAPSQ) fixation was performed by using the ilioinguinal approach. Postoperative X-ray (**d**) showed good fracture reduction. Postoperative 3D CT reconstruction showed residual displacement was less than 1 mm and graded as excellent according to classic Matta criteria. **e** The anterior inclined angle of quadrilateral plate was 86.90°; **f** The middle inclined angle of quadrilateral plate was 92.19°; **g** The posterior inclined angle of quadrilateral plate was 104.69°. All the three inclined angles were not within normal range, and rated as poor according to evaluation criteria of inclined angles. Combined with the classic Matta criteria, the modified Matta criteria of this patient graded as good. The Harris hip score of the last follow-up was 75, graded as fair

As depicted in Tables 4 and 5, among the cases evaluated as excellent or good according to the classic Matta criteria, the excellent-to-good rates of hip function were 70.3% and 61.1%, respectively. Among the cases evaluated as poor according to the classic Matta criteria, the fair-to-poor rate of hip function was 50% (Table 6). In contrast, among the cases evaluated as excellent or good according to the modified Matta criteria, the excellent-to-good rates of hip function were 78.4% and 73.7%, respectively. Among the cases evaluated as poor according to the modified Matta criteria, the fair-to-poor rate of hip function was 75%. Differences between all the three groups were statistically significant (*p* < 0.05). A typical case is shown in Fig. 3.

**Table 4:** Comparison of Harris functional grading among the excellent cases of classic Matta criteria and modified Matta criteria

Group	N	Harris functional grading				Excellent and good rate
		Excellent	Good	Fair	Poor	
Classic Matta criteria	101	51 (50.5%)	20 (19.8%)	19 (18.8%)	11 (10.9%)	70.3%
Modified Matta criteria	37	24 (64.9%)	5 (13.5%)	4 (10.8%)	4 (10.8%)	78.4%
*Chi-square* value						5.88
*p*-value						*p* < 0.05

**Table 5:** Comparison of Harris functional grading among the good cases of classic Matta criteria and modified Matta criteria

Group	N	Harris functional grading				Excellent and good rate
		Excellent	Good	Fair	Poor	
Classic Matta criteria	18	7 (38.9%)	4 (22.2%)	4 (22.2%)	3 (16.7%)	61.6%
Modified Matta criteria	76	29 (38.2%)	27 (35.5%)	11 (14.5%)	9 (11.8%)	73.7%
*Chi-square* value						5.13
*p*-value						*p* < 0.05

**Table 6:** Comparison of Harris functional grading among the poor cases of classic Matta criteria and modified Matta criteria

Group	N	Harris functional grading				Fair and poor rate
		Excellent	Good	Fair	Poor	
Classic Matta criteria	6	1 (16.7%)	2 (33.3%)	1 (16.7%)	2 (33.3%)	50%
Modified Matta criteria	12	2 (16.7%)	1 (8.3%)	2 (16.7%)	7 (58.3%)	75%
*Chi-square* value						6.08
*p*-value						*p* < 0.05

## Discussion

The acetabular quadrilateral plate is a square area on the medial side of the acetabulum. Based on the definition of the quadrilateral plate by previous researchers, we collectively refer to the relatively flat area that below the pelvic boundary line and above the level of the ischial spine as the quadrilateral plate [14, 15]. There is a certain degree of inclination of the acetabular quadrilateral plate relative to the transverse plane of the pelvis, which is named the inclined angle of acetabular quadrilateral plate by our group. So far, no literature has reported the naming of this angle. Letournel and Judet classification did not classify quadrilateral plate fractures, and except for simple anterior and posterior wall fractures, the vast majority of acetabular fractures involve the quadrilateral area [8]. Some scholars have analyzed the fracture lines of the quadrilateral plate through CT 3D reconstruction, explored the relationship between fracture lines in this place and the Judet-Letournel classification of acetabular fractures [14].

Anatomic and stable reduction in quadrilateral plate fractures is of paramount importance for obtaining a reasonable functional outcome [16, 17]. Currently, the commonly used postoperative imaging evaluation for fracture reduction is the Matta evaluation criteria, which measures the fracture residual displacement distance to determine its quality [3]. However, it has found that some cases that met the Matta criteria for excellent reduction did not have corresponding excellent functional recovery. Additionally, several studies also measured the step displacement of the fracture site on X-ray or CT and took the maximum value as the reference to evaluate the reduction quality [18]. Their findings also showed that step displacement did not correlate with the function of the affected hip. These results indicated that the Matta evaluation criteria themselves have shortcomings. In this study, among the 101 excellent cases evaluated by classic Matta criteria, there are 37, 34, and 30 cases rating as excellent, good, and poor, respectively, according to the evaluation criteria of the inclined angles. As for 18 good cases, 6, 5, and 7 cases rating as excellent, good and poor, respectively. And in terms of the 6 poor cases, 1, 2, and 3 cases rating as excellent, good and poor, respectively. This result suggested that even in the cases that class Matta criteria graded as excellent or good, there were still many cases existing issues on inclined angles after surgery. To our understanding, this evaluation criteria only consider the issue of fracture alignment. As acetabulum is a complex and irregular structure, the rotation of the fracture fragments cannot be evaluated, which is a significant defect of Matta criteria. Currently, there are no relevant evaluation criteria for the rotation displacement of the acetabular plate. Therefore, in view of the clinical need, it is essential to identify indicators that could evaluate the rotation displacement after acetabular fracture surgery, thus improve the postoperative imaging evaluation of acetabular fractures.

Due to the extremely complex structure of the acetabulum, it is unrealistic to perform 3D quantitative analysis of its morphology under gross anatomy or X-ray images. In contrast, the structure of the medial area is relatively flat, so quadrilateral plate may indirectly reflect acetabular fracture reduction by studying this area [11]. The greater sciatic notch, sciatic spine, obturator foramen and pelvic boundary were usually used as marks to describe and locate the quadrilateral plate. In this study, with reference to bony landmarks such as anterior and posterior margins of the acetabulum, ischial spine, ischial tuberosity, and posterior margin of the obturator foramen, the quadrilateral plate is divided by "3 lines" including the anterior, middle and posterior inclination lines. The anterior, middle and posterior inclination lines can represent the general trend and anatomical characteristics of the quadrilateral plate. The pelvic boundary is arc-shaped structure, the posterior line starts from the posterior end of the arc, and the midline is the vertical bisector of the arc, dividing the quadrilateral plate into anterior and posterior zones. The division of the quadrilateral plate into two zones is able to accurately indicate the rotation displacement of the acetabulum. Rotated displacement of the quadrilateral plate will inevitably lead to position changes of the anterior, middle or posterior inclination lines. And the changes of two or more inclined lines/angles could indicate the rotation of the quadrilateral plate. For example, if the anterior and middle inclined angles are abnormal while the posterior inclined angle is normal, it means the alignment of the anterior half of the quadrilateral plate is abnormal while the alignment of the posterior part is good. Conversely, if the posterior inclination angle is abnormal while the anterior and middle inclined angles are normal, it suggests that the posterior part is abnormal and the alignment of the front part is good. If all three inclined angles are abnormal, both the anterior and posterior halves of the quadrilateral plate will show abnormal alignment, indicating rotation of the acetabulum. The Matta criteria lack the awareness of rotation evaluation and this study complements and improves the evaluation of postoperative acetabular plate fractures.

In present study, we have used the Mimics digital software to measure the average values of the three angles and also compared the difference between male and female. Our result showed that whether in males or in females, the anterior, middle, and posterior inclined angles showed a progressively increasing trend. Meanwhile, all the three inclined angles in males were all significantly larger than those in females. This result could be related to the special physiological structure between males and females. The quadrilateral plate on both sides forms the lateral wall of the pelvic cavity. The male pelvic cavity is funnel-shaped, while the female pelvic cavity is barrel-shaped. The measurement data from this study further confirms this. In addition, this investigation used CT data from health adults. If cadaveric pelvic specimens are chosen as the subject of this study, there are many disadvantages such as inaccurate positioning, poor repeatability, angular measurement errors, and high costs [20]. 3D reconstruction of pelvis could not only measure the distance between fracture ends, but also various angles at different positions though the function of rotation or cutting in Mimics. Moreover, several special anatomical structures such as the large sciatic notch, sciatic tuberosity, sciatic spine, pelvic boundary, and closed foramen were selected as reference markers for measurement. These anatomical structures are easily recognized intraoperatively. Therefore, our group will design special angle measurer for intraoperative use to further guide fracture reduction in future.

Apart from that, we also compared the results of classic Matta criteria and our modified Matta criteria in clinical use. Our results showed that among the cases evaluated as excellent according to the classic Matta criteria, the excellent-to-good rates of hip function were 70.3%. In contrast, among the cases evaluated as excellent according to the modified Matta criteria, the excellent-to-good rates of hip function were 78.4%. Among the cases evaluated as poor according to the modified Matta criteria, the fair-to-poor rate of hip function was 75%, while for classic Matta criteria, this value was 50%. As can be seen, cases with hip function rated as excellent and good were randomly distributed among cases evaluated as excellent, good, and poor graded by classic Matta criteria, whereas they were distributed concentrically among cases evaluated as excellent and good in modified Matta criteria. In addition, cases with hip function rated as fair and poor were randomly distributed among cases evaluated as excellent, good, and poor graded by classic Matta criteria, whereas they were distributed concentrically among cases evaluated as fair and poor in modified Matta criteria. The results indicated that our modified Matta criteria could accurately predict the functional recovery of the hip in addition to correctly assessing the quality of fracture reduction. The changes of inclined angles, especially in the middle inclined angle, could inevitably lead to changes in the acetabular structure or position. The change of acetabular structure is mainly manifested as step displacement and gap displacement. Previous studies showed that gap displacement was not correlated with hip function, whereas the larger the step displacement, the poorer the hip function [18]. In addition, the change of change of position are mainly manifested as asymmetry of bilateral acetabulum, failure to restore the concentric structure between the femoral head and acetabulum, which may cause a change in the physiological force line of the lower limb and accelerate the degenerative changes in the affected-side hip or even knee joint [21].

## Limitations

Although this study is the first to present the concept of inclined angle of acetabular quadrilateral plate, it should be noted that there are still some limitations. Firstly, the health sample size for the measurement of normal inclined angles was small, and there was a possibility of type II statistical error. Therefore, future research with larger sample sizes is needed. Secondly, in this study, although the postoperative imaging evaluation of acetabular plate fracture was analyzed in depth, it should be further discussed whether there is a closer relationship between the postoperative imaging evaluation of acetabular plate fracture and patients’ functional outcomes. In our experience, achieving good functional results after acetabular fracture not only lies in the anatomical repositioning of the acetabulum, but also requires patients to adhere to a reasonable rehabilitation after surgery [22]. Thirdly, in spite of the simplifications in the measurement of these three angles, the measurement steps are still complex, which may hinder clinical promotion and popularization. Thus, further improvement and optimization of this method are needed in further studies. Moreover, this study did not involve the measurement of inclined angles in pediatric and geriatric populations. In order to gain a more comprehensive understanding of the variations of these anatomical parameters in different age groups, future research will focus on exploring this area, particularly in relation to pediatric and geriatric patients.

## Conclusions

There exist inclined angles in the acetabular quadrilateral plate and they have gender differences. Inclined angles of the quadrilateral plate could be used to assess the quality of fracture reduction and provide a basis for evaluating the rotational displacement of fracture blocks in the quadrilateral plate, which compensates the shortage of classic Matta criteria. Moreover, the modified Matta criterion was more accurate in assessing the postoperative functional recovery in patients with acetabular plate fractures, and further confirmed the feasibility of using inclined angles for the evaluation of the fracture reduction quality. In addition, the modified Matta criteria could also provide important reference information for the postoperative plans of rehabilitation exercise of acetabular fracture patients, and ultimately achieve the goal of improving clinical outcomes.

## Data Availability

The dataset used in the current study are available from the corresponding author on a reasonable request.
