# Correction: Inclined angles of acetabular quadrilateral plate: digital measurement and clinical application of the new anatomical concept

**DOI:** 10.1186/s13018-023-04459-0

**Published:** 2024-01-08

**Authors:** Xiaofeng Chen, Haiyang Wu, Kunming Cheng, Ximing Liu, Xianhua Cai

**Affiliations:** 1 Department of Orthopaedic Surgery, Yangxin People’s Hospital, Yangxin, 435200 Hubei China; 2 Department of Clinical Medicine, Graduate School of Tianjin Medical University, Tianjin, 301700 China; 3 Department of Intensive Care Unit, The Second Afliated Hospital of Zhengzhou University, Zhengzhou, Henan China; 4 Department of Orthopaedic Surgery, General Hospital of Central Theater Command, Wuhan, 430064 Hubei China; 5 Department of Orthopaedic, South China Hospital of Shenzhen University, Shenzhen, 518116 Guangdong China

**Correction : Journal of Orthopaedic Surgery and Research (2023) 18:723** 10.1186/s13018-023-04143-3

In this article the affiliation details for Author “Xianhua Cai were incorrectly given as “Department of Orthopaedic Surgery, South China Hospital Afliated to Shenzhen University, Shenzhen 518111, Guangdong, China “ but should have been “Department of Orthopaedics, South China Hospital of Shenzhen University, Shenzhen, 518116, P. R. China”.
